# Variable Absorption of Clavulanic Acid After an Oral Dose of 25 mg/kg of Clavubactin® and Synulox® in Healthy Cats

**DOI:** 10.1100/tsw.2002.287

**Published:** 2002-05-21

**Authors:** Tom B. Vree, Eric Dammers, Eri van Duuren

**Affiliations:** Institute for Anaesthesiology, University Medical Center Sint Radboud, P.O. Box 9101, 6500 HB Nijmegen, The Netherlands; DADA Consultancy, Dennenstraat 109, 6543 JR Nijmegen, The Netherlands; Regivet BV, Boswinde 113, 2496 WG Den Haag, The Netherlands

**Keywords:** absorption, amoxicillin, cats, clavubactin, clavulanic acid, inhibition, variable AUC

## Abstract

The aims of this investigation were to calculate the pharmacokinetic parameters and to identify parameters, based on individual plasma concentration-time curves of amoxicillin and clavulanic acid in cats, that may govern the observed differences in absorption of both drugs. The evaluation was based on the data from plasma concentration-time curves obtained following a single-dose, open, randomised, two-way crossover phase-I study, each involving 24 female cats treated with two Amoxi-Clav formulations (formulation A was Clavubactin® and formulation was B Synulox® ; 80/20 mg, 24 animals, 48 drug administrations). Plasma amoxicillin and clavulanic acid concentrations were determined using validated bioassay methods. The half-life of elimination of amoxicillin is 1.2 h (t_1/2_ = 1.24 ± 0.28 h, C_max_ = 12.8 ± 2.12 μg/ml), and that of clavulanic acid 0.6 h (t_1/2_ = 0.63 ± 0.16 h, C_max_ = 4.60 ± 1.68 μg/ml). There is a ninefold variation in the AUC_t_ of clavulanic acid for both formulations, while the AUC_t_ of amoxicillin varies by a factor of two. The highest clavulanic acid AUC_t_ values indicate the best absorption; all other data indicate less absorption. Taking into account that the amoxicillin–to–clavulanic acid dose ratio in the two products tested was 4:1, the blood concentration ratios may actually vary much more, apparently without compromising the products’ high efficacy against susceptible microorganisms.

### INTRODUCTION

Clavulanic acid broadens the antibacterial spectrum of amoxicillin by rendering β-lactamase–producing strains susceptible to amoxicillin[1]. The binding of β-lactamases with clavulanic acid is a complex physicochemical process. In general, the initially formed complex is rapidly depleted as covalent binding leads to the irreversible inactivation of the β-lactamase and clavulanic acid. Clavulanic acid has thus been termed a 'suicide' inhibitor for β-lactamases. As a result of this inhibition, compounds such as amoxicillin, which would normally have been hydrolysed to an extent of 10–20% and bound by β-lactamase, are spared. As both amoxicillin and clavulanic acid are both absorbed after oral administration and possess similar pharmacokinetic properties, they offer a rational antimicrobial combination[2,3,4,5,6].

In humans, the standard dose of amoxicillin/clavulanic acid for adults with lower respiratory tract infection has, for many years, been 500/125 mg orally t.i.d. A fixed dosage ratio of amoxicillin/clavulanic acid (in humans 4:1 or 7:1), assumes that there is little variation in the absorption of the drug from the pharmaceutical formulation and that the plasma concentration ratio, and the respective AUC values of both compounds, are more or less constant[3,5]. However, in humans, bioequivalence studies with the drug ratios 500/125 and 875/125 mg have demonstrated that clavulanic acid shows unpredictable absorption and AUC values, hence challenging the principle of the fixed-dose ratio (Vree et al., personal observation).

The pharmacokinetics of amoxicillin and clavulanic acid in cats are not well known. As both drugs are eliminated via renal excretion, it is speculated that the drugs would show similar pharmacokinetic properties to those in humans.

This pharmacokinetic evaluation is a follow-up to a bioequivalence study in which two 40/10-mg tablets (25/6.25 mg/kg) Clavubactin® formulations administered to cats showed bioequivalence despite a ninefold variation in the $AUC_t$ values of clavulanic acid[7].

The aims of this study were to describe the plasma concentration-time curves of amoxicillin and clavulanic acid in cats, to calculate pharmacokinetic parameters, and to identify parameters that may govern the differences in release and absorption of both drugs.

### MATERIALS AND METHODS

#### Animals

Twenty-four adult female cats (strain Hsd/Cpb: CADS) were obtained from Harlan Winkelman (D33178 Borchen, Germany). Three cats were housed in one kennel; the room temperature was kept at 19.5 ± 3.5$^{o}$C and relative humidity was kept between 40 and 80%.The animals received cat food 4MM, 0.9 MRAD (Hope Farm, 3440 AB Woerden, the Netherlands).

#### Experimental Design

The evaluation was based on the data from plasma concentration-time curves obtained from a single-dose, open, randomised, two-way, crossover phase-I study, each involving 24 female cats treated with two Amoxi-Clav formulations (24 cats, 48 drug administrations). Treatments were separated by a 3-week washout period.

The clinical trials were performed by Harlan Bioservice (Walsrode, D-29664 Germany) in accordance with the requirements of the guidelines: EEC Council directives 81/852/EEG, 28-09-1981, and 92/18/EEC 20-03-1992. The study protocols were granted approval by the Institutional Animal Ethics committee of Harlan.

### Trial Course

For each study, the animals were divided randomly into two groups. Randomisation was carried out by procedure PLAN of the SAS Institute (Cary, NC). Group 1 was assigned to treatment sequence I–II (formulation I-II). Group 2 was assigned to sequence II–I. During the two crossover sessions, cats received each of the two formulations following an overnight fast:

- Formulation A = single oral dose of two Clavubactin® 40/10-mg tablets (Le Vet BV, Oudewater, the Netherlands); 50 mg amoxicillin trihydrate and 12.5 mg potassium salt of clavulanic acid; mg based on free base);
- Formulation B = single oral dose of two Synulox® 50 mg, (Pfizer, Capelle aan de IJssel, the Netherlands). The Clavubactin® tablets were made 20% smaller (40/10 mg) to match the Synulox® tablets.

The 24 healthy female domesticated cats without any comedication (body weight 3.14 ± 0.40 kg, range 2.50–3.76 kg) received 26.2 ± 3.1 mg/kg of amoxicillin and 6.55 ± 0.77 mg/kg of clavulanic acid (target dose of 25/6.25 mg).

### Drugs

- Amoxicillin: [2S-[2α,5α,6β(S*)]]-6-[[amino(4-hydroxyphenyl)acetyl]amino]-3,3-dimethyl-7-oxo-4-thia-1-azabicyclo[3.2.0]heptane-2-carboxylic acid. $C_{16}H_{19}N_3O_5S$, MW 365.41, $[\alpha]^{20}_D$ = +246°, CAS 26787-78-0. Trihydrate, CAS 61336-70-7.
- Clavulanic acid : (Z)-(2R,5R)-3-(2-hydroxyethylidene)-7-oxo-4-oxa-1-Azabicyclo [3.2.0]heptane-2carboxylic acid. $C_8H_9NO_5$, MW 199.16, CAS 58001-44-8.

### Drug Administration

Before drug administration, the cats fasted for at least 10 h. Fasting was continued until 4 h after dosing. On the day of the experiment, food was offered for about 30 min at 4 h after drug administration. All other days, food was offered *ad libitum* at about 09.00 h for about 30 min/day. Tap water was continuously available ad libitum via bowls.

The tablets were administered to the animals on the base of the tongue. Together with the tablets, each animal received a small volume of water in order to assure the correct intake.

One cat was too aggressive and refused to blood sampling, thus leaving 23 cats for evaluation.

### Blood Sampling

Blood samples (3 ml) were withdrawn from the jugular vein using a needle No. 14, 23G × 1.25" (0.6 × 30mm). They were collected in heparinised glass tubes just before dosing, and at 0.25, 0.50, 0.75, 1, 1.3, 1.6, 2, 2.5, 3, 4, 5, 7, and 10 h after dosing. The blood samples were centrifuged at 3,500 rpm for 10 min and plasma was separated.

### Sample Processing

The plasma samples were divided into two analysis samples by transferring them into polypropylene tubes. Subsequently the samples were diluted 9+1 with two different phosphate buffers: for amoxicillin, the samples were diluted with 1-M potassium phosphate buffer; for clavulanic acid the samples were diluted with a 1-M sodium phosphate buffer. The samples were stored at –70ºC until analysed.

### Bioanalysis

Plasma amoxicillin and clavulanic acid concentrations were determined using a validated bioassay (FarmaResearch, Nijmegen, the Netherlands). Lower limit of quantitation (LOQ) values were 10 ng/ml for both drugs. The average amoxicillin recovery from cat plasma was 92.3 ± 6.46% and for clavulanic acid 95.8 ± 7.93%.

For the spiked quality control standards of amoxicillin, the interday precision ranged from 1.42–0.24% CV (low-high QC samples). The intraday precision was 1.27–1.07% CV (low-high QC samples). For the spiked quality control standards of clavulanic acid, the interday precision ranged from 1.49–0.44% CV (low-high QC samples). The intraday precision of the clavulanic assay ranged from 1.19–0.88% CV (low-high QC samples). There was no interference between both drugs on each assay.

### Pharmacokinetics

Based on the plasma amoxicillin and clavulanic acid concentrations of individual animals, the following pharmacokinetic parameters were determined by noncompartmental analysis using SAS 6.12 (SAS Institute Inc., 1989): maximum plasma drug concentration ($C_{max}$: μg/ml); time to reach $C_{max}$ ($t_{max}$, h); area under the plasma concentration-time curve up to the last measurable concentration ($C_t$), calculated by the linear trapezoidal method ($AUC_t$, μg.h/ml); $AUC_t$/kg is the $AUC_t$ corrected for the body weight of the individual animal; elimination half-life associated with the terminal slope of a semilogarithmic concentration-time curve ($t_{1/2z}$, h), calculated as $\ln 2/\lambda_z$, where λ is the elimination rate constant.

### Statistical Analysis

Analysis of variance (ANOVA) was carried out, and significance was defined at $p \leq 0.05$.

## RESULTS

The mean plasma concentration-time curves of amoxicillin and clavulanic acid in 23 healthy female cats after oral administration of two 40/10-mg Clavubactin® tablets are shown in Fig. 1a. The half-life of elimination of amoxicillin is 1.2 h ($t_{1/2}$ 1.24 ± 0.28 h, $C_{max}$ 12.8 ± 2.12 μg/ml), and that of clavulanic acid is 0.6 h ($t_{1/2}$ 0.63 ± 0.16 h, $C_{max}$ 4.60 ± 1.68 μg/ml). Fig. 1b shows the pharmacokinetic profile of Synulox®. Some pharmacokinetic parameters for both drugs are summarised in Table 1. The two formulations were bioequivalent.

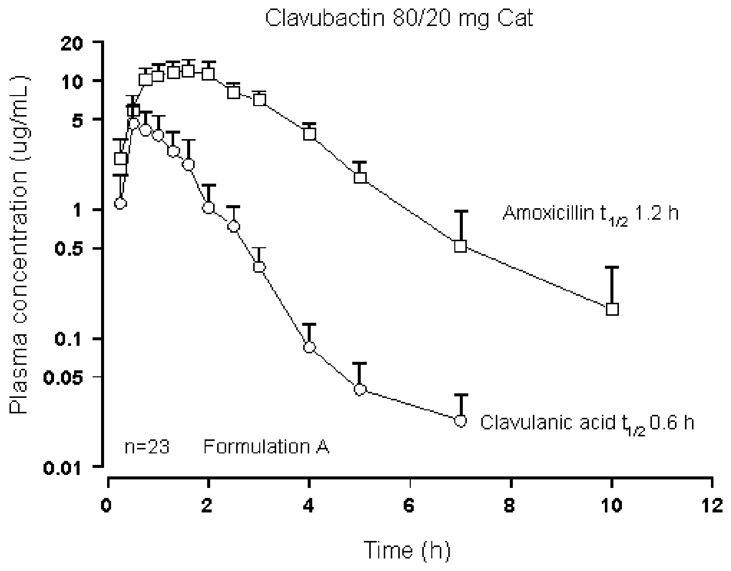

**FIGURE 1a.** Mean plasma concentration-time curves of amoxicillin (squares) and clavulanic acid (dots) after an oral dose of 26/6 mg/kg Clavubactin® to 24 healthy female cats (A formulation). The $t_{1/2}$ of the mean amoxicillin curve is 1.2 h (1.24 ± 0.28 h, n = 23) and that of clavulanic acid is 0.6 h (0.63 ± 0.16 h, n = 23).

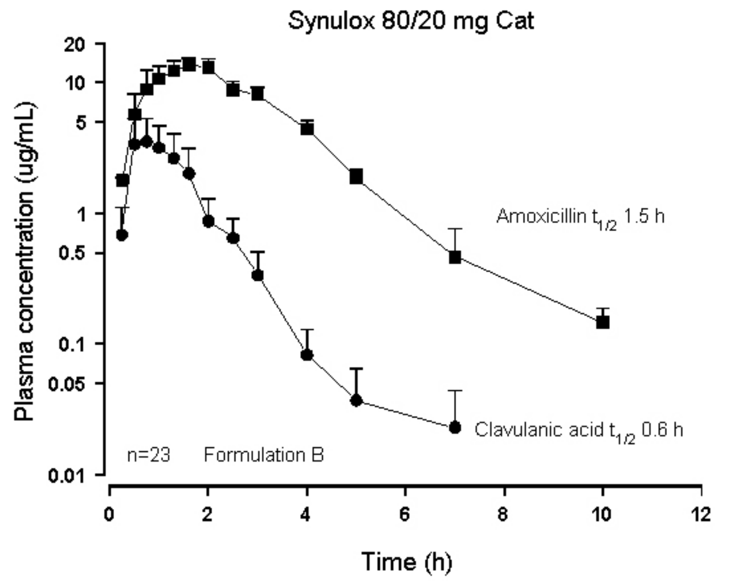

**FIGURE 1b.** Mean plasma concentration-time curves of amoxicillin and clavulanic acid after an oral dose of 26/6 mg/kg Synulox® to 23 healthy female cats (B formulation). The $t_{1/2}$ of the mean amoxicillin curve is 1.5 h (1.51 ± 0.21 h, n = 23) and that of clavulanic acid 0.6 h (0.61 ± 0.16 h, n = 23).

Fig. 2 shows a plot of the $AUC_{t\ amoxicillin}$ vs. the $AUC_{t\ clavulanic\ acid}$ after oral administration of two 40/10-mg Clavubactin® tablets of both formulations. There is a ninefold variation in the $AUC_t$ of clavulanic acid for both formulations (range 13.3–1.45 µg.h/ml), while the corresponding $AUC_t$ of amoxicillin varies by a factor of two. The mean ratio of the $AUC_t$ amoxicillin/clavulanic acid is 7.32 ± 3.49 for formulation A and 9.45±5.60 for formulation B ($p$ = 0.13, Table 1).

**TABLE 1**
**Some Pharmacokinetic Variables of Amoxicillin (80 mg) and Clavulanic Acid (20 mg) in Female Cats (n = 23, Body Weight 3.14 ± 0.40 kg)**

| | Amoxicillin | | Clavulanic Acid | |
|---|---|---|---|---|
| | A | B | A | B |
| Dosage mg/kg | 26.2 ± 3.07 | 26.0 ± 3.11 | 6.55 ± 0.77 | 6.86 ± 0.81 |
| $C_{max}$ (µg/ml) | 12.8 ± 2.12 | 12.0 ± 3.12 | 4.60 ± 1.68 | 3.84 ± 1.82 |
| $t_{max}$ (h) | 1.47 ± 0.44 | 1.57 ± 0.43 | 0.72 ± 0.26 | 0.81 ± 0.23 |
| $t_{1/2}$ (h) | 1.24 ± 0.28 | 1.51 ± 0.21 | 0.63 ± 0.16 | 0.61 ± 0.16 |
| $AUC_t$ µg.h/ml | 38.4 ± 4.64 | 38.1 ± 7.85 | 6.16 ± 2.19 | 5.49 ± 2.67 |
| $AUC_t$/kg | 12.4 ± 2.14 | 12.7 ± 3.65 | 2.00 ± 0.77 | 1.73 ± 1.02 |
| Ratio $AUC_t$ | 7.32 ± 3.49 | 9.45 ± 5.60 | | |
| p | 0.13 | | | |

*Note*: A = formulation A, Clavubactin®; B = formulation B, Synulox®; Ratio $AUC_t$ amoxicillin/clavulanic acid (=P); $AUC_t$/kg = $AUC_t$ /bodyweight (µg.h/ml/kg)

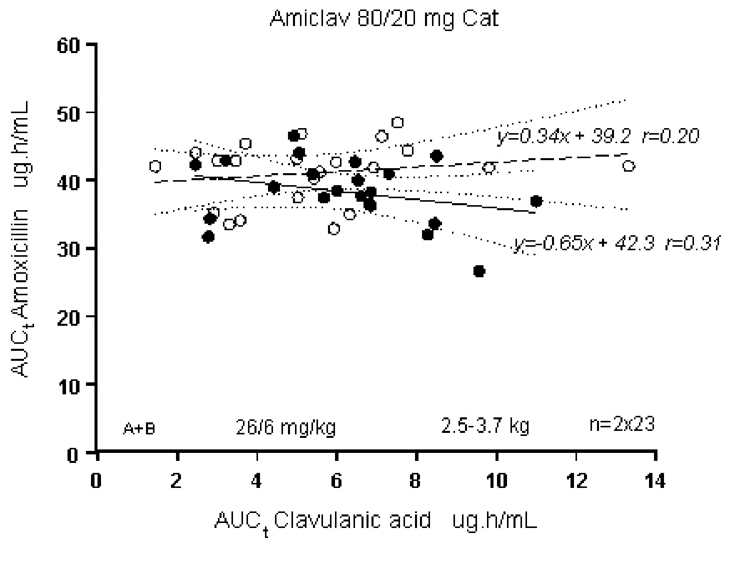

**FIGURE 2.** Individual $AUC_t$ values of clavulanic acid plotted vs. the corresponding $AUC_t$ amoxicillin values in 2 × 23 female cats. Solid dots = formulation A, open dots = formulation B. It can be noticed that there is a ninefold variation in the $AUC_t$ values of clavulanic acid, while the variation for amoxicillin is less (2×). Dotted lines represent the 95% confidence interval.

Fig. 3 shows a plot of the $AUC_{t\ amoxicillin}$ vs. the $AUC_{t\ clavulanic\ acid}$ after oral administration of two 40/10-mg Clavubactin® tablets. The $AUC_t$ values are corrected for the body weight of the individual cats. The mean $AUC_t$/kg values for amoxicillin are 12.4 ± 2.14 vs. 12.7 ± 3.65 µg.h/ml/kg for formulations A and B ($p$ = 0.55), respectively, and for clavulanic acid 2.00 ± 0.77 vs. 1.73 ± 1.02 µg.h/ml/kg ($p$ = 0.53, Table 1).

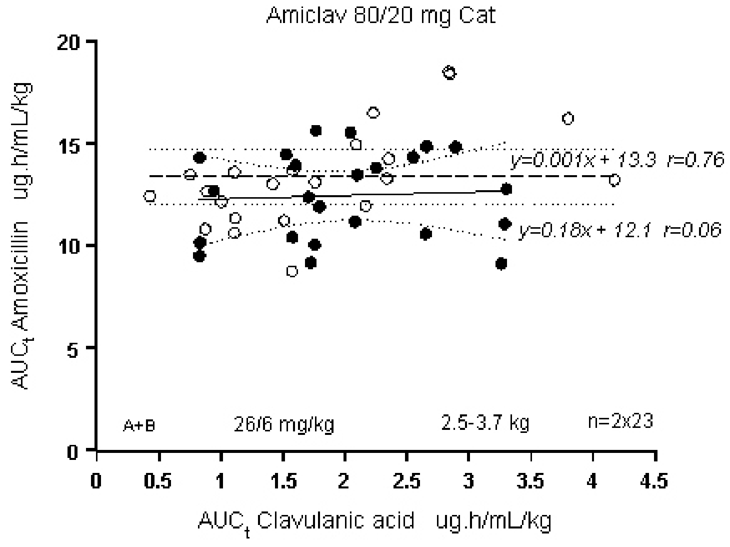

**FIGURE 3.** Individual AUC$_t$ values of clavulanic acid corrected for the body weight (kg) plotted vs. the corresponding AUC$_t$ amoxicillin values in 2 × 23 cats. Solid dots = formulation A, open dots = formulation B. It can be noticed that there is a ninefold variation in the AUC$_t$ values of clavulanic acid, while the variation for amoxicillin is twofold. Dotted lines represent the 95% confidence interval.

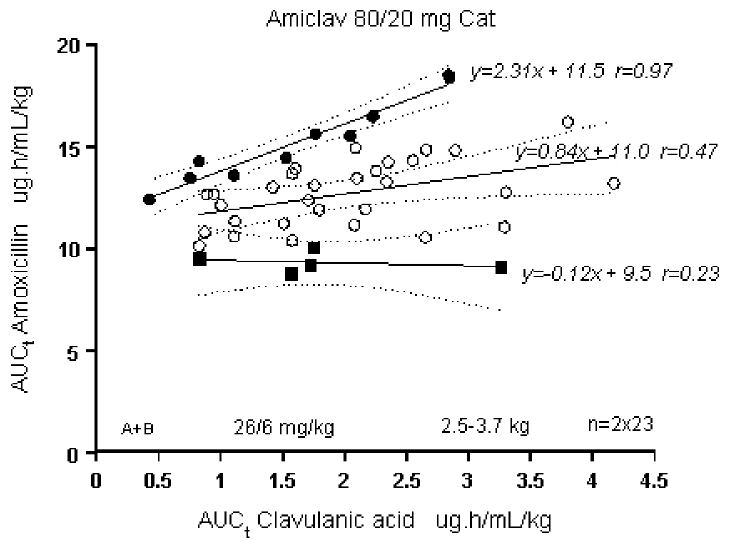

**FIGURE 4.** Differentiation between the AUC$_t$/kg values in three apparent subgroups. Dotted lines represent the 95% confidence interval.

Fig. 4 shows the whole data set of AUC$_t$/kg values of both formulations in 2 × 23 cats (Fig. 3). Three apparent groups of data can be distinguished, each characterised by a regression line.

An upper tangent ($y = 2.31x + 11.47$; $r = 0.97$), a lower tangent ($y = -0.12x + 9.5$; $r = 0.23$), and an intermediate group ($y = 0.84x + 11.0$, $r = 0.47$) of data can be distinguished. Both the slopes and the y-intercepts of the three apparent data groups (high-medium-low) are statistically different. The statistical differences between the slopes are high-medium, $p < 0.0001$, high-medium, $p < 0.0001$; high-low, $p < 0.0001$. The statistical differences between the y-intercepts are high-medium, $p = 0.0308$, high-medium, $p < 0.0001$; high-low, $p < 0.0001$.

### DISCUSSION

In the animals, the distribution of the data with the Clavubactin® and Synulox® formulations is at random. The clavulanic acid $AUC_t$ data range from 0.5–4.5 µg.h/ml/kg, i.e, by a factor of nine. This variation in $AUC_t$ is not seen when clavulanic acid was administered alone[8,9]. When the $AUC_t$ values were corrected for the body weight of the cats, the picture in Figs. 3 and 4 shows clear apparent upper and lower values in the $AUC_t$ values. In humans, with high dosage amoxicillin 875 mg (≈10 mg/kg), the $C_{max}$ broadens, indicating a rate-limiting step in the absorption process[10,11,12,13,14,15]. Figs. 3 and 4 suggest that there are three apparent groups or situations demonstrating a saturable absorption of amoxicillin (high y-intercept) due to the high dosage of 26 mg/kg. While there is still an increase in the $AUC_t$ of clavulanic acid, the $AUC_t$ of amoxicillin in each of the three apparent groups reaches a plateau value or increases only slightly. The $t_{1/2}$ amoxicillin is twice that of clavulanic acid, which may indicate extended absorption due to the high dose (Table 1). Normally the elimination $t_{1/2}$ of both drugs is similar[16,17,18,19].

With dogs, a similar behaviour was observed, but initially there was a clear $F$.Dose-$AUC_t$ relationship for both amoxicillin and clavulanic acid, before a plateau level was reached. It seems that in cats a plateau level in the relation $AUC_t$ amoxicillin/clavulanic acid was reached much earlier, showing the flat slopes and the high y-intercept and $AUC_t$ of amoxicillin[20].

The 26-mg/kg amoxicillin dose must show capacity-limited absorption like in humans. In the ratio $AUC_t$ amoxicillin/clavulanic acid high ratios can be obtained with a decrease in the denominator. This implies that the high dose of amoxicillin inhibits the absorption of clavulanic acid.

The highest $AUC_t$ clavulanic acid values indicate the greatest extent of absorption; all other data indicate lesser absorption or a higher rate of elimination by metabolism or hydrolysis. All cats were healthy, i.e., presumably with a low and constant amount of bacteria containing β-lactamase activity. Although the mean plasma concentration-time curves of clavulanic acid in both formulations suggest comparable absorption, the variation in the individual absorptions amounts a factor of nine, like in humans[21,22,23].

#### Amoxicillin-Clavulanic Acid Dosage Ratio

The amoxicillin-clavulanic acid dosage ratio is based on clavulanic acid irreversibly binding to β-lactamases. When 80% of an oral amoxicillin dose is excreted unchanged in the urine, 20% remains for enzymatic degradation[24,25,26]. The dosage ratio of 4:1 is understandable; however, the total amount of bacteria with β-lactamase activity in each animal or human patient is unknown.

In humans, a dose ratio of 10:1 (50/5 mg/kg) has been used for intravenous infusion (0.5 h), which resulted in a $AUC_t$ ratio of 10 and an identical plasma clearance[21]. In goats, an intravenous dose ratio of 20/5 mg/kg resulted in an $AUC_t$ ratio of 4.4 and an identical plasma clearance [16].

### Principal Parameters for the $AUC_t$

The variation in $AUC_t$ amoxicillin is twofold, while that of clavulanic acid is ninefold. The regression lines of the three apparent subgroups show a high y-intercept of approximately 10 $AUC_t$ amoxicillin units (Fig. 4). This suggests that with $AUC_t$ clavulanic acid $\lim X \rightarrow 0$ there is still substantial $AUC_t$ amoxicillin, thus substantial absorption, while there is no clavulanic acid present. This can be explained assuming that amoxicillin is first absorbed, or transported by the capacity limited transport mechanism into the general circulation, and that thereafter, depending on the free transport capacity clavulanic acid will be absorbed[27]. If sequential absorption would be the case, then there would be a difference in $t_{max}$ between amoxicillin and clavulanic acid. This is present, though the $t_{max}$ clavulanic acid is only 50% of that of amoxicillin ($t_{max}$ = 1.47 ± 0.44 h, 1.57 ± 0.43 vs. 0.72 ± 0.26 h, 0.81 ± 0.23 h, respectively, in Table 1). Most likely, amoxicillin and clavulanic acid start to be absorbed at the same moment, while after a while clavulanic acid absorption will be limited or inhibited by amoxicillin, leading to the smaller $t_{max}$.

### CONCLUSION

From this study it is learned that the 25/6.25-mg/kg (80/20-mg) dose gives a ninefold variation in the absorption or in the final $AUC_t$ value of clavulanic acid. The high dose of amoxicillin may interfere with or inhibit the absorption of clavulanic acid. This indicates that even (animal) patients with a resulting 1:36 $AUC_t$ ratio may or must benefit from the presence of clavulanic acid. Clinical efficacy will be reached by an apparent overly high proportion of clavulanic acid in fixed-dose combination preparations.
